# Aqua[1-(pyridin-2-yl)ethanone oximato][1-(2-pyridin-2-yl)ethanone oxime]­copper(II) perchlorate mono­hydrate

**DOI:** 10.1107/S1600536812023872

**Published:** 2012-06-13

**Authors:** Baoyun Zhong, Shengli Li, Guifang Chen

**Affiliations:** a The Third Middle School in Liaocheng, Shandong 252059, People’s Republic of China; b Department of Chemistry and Biology, Dongchang College Liaocheng University, Shandong 252059, People’s Republic of China

## Abstract

In the title compound, [Cu(C_7_H_7_N_2_O)(C_7_H_8_N_2_O)(H_2_O)]ClO_4_·H_2_O, the Cu^II^ ion is five-coordinated by the N atoms from the 1-(pyridin-2-yl)ethanone oximate and 1-(pyridin-2-yl)ethan­one oxime ligands and by the water O atom in a distorted square-pyramidal geometry. The two organic ligands are linked by an intra­molecular O—H⋯O hydrogen bond. In the crystal, mol­ecules and ions are linked by O—H⋯O hydrogen-bonding inter­actions, forming chains along the *a* axis. The perchlorate O atoms are disordered in a 0.58 (2):0.42 (2) ratio.

## Related literature

For the coordination chemistry of oximes, see: Chaudhuri (2003 ▶); Pavlishchuk *et al.* (2003 ▶). For related structures, see: Qiu *et al.* (2011 ▶); Wu & Wu (2008 ▶); Zuo *et al.* (2007 ▶). For the properties of related complexes, see: Davidson *et al.* (2007 ▶); Clerac *et al.* (2002 ▶). 
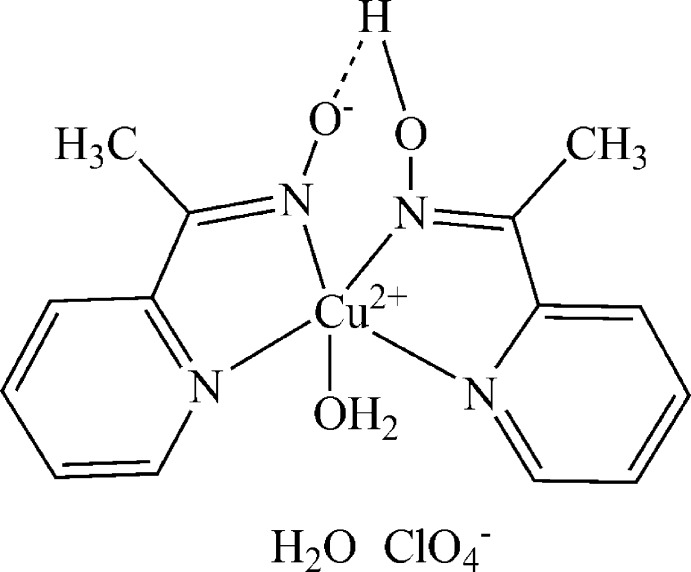


## Experimental

### 

#### Crystal data

[Cu(C_7_H_7_N_2_O)(C_7_H_8_N_2_O)(H_2_O)]ClO_4_·H_2_O
*M* *_r_* = 470.32
Monoclinic, 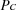

*a* = 6.3526 (7) Å
*b* = 15.7199 (14) Å
*c* = 9.8235 (9) Å
β = 101.235 (1)°
*V* = 962.20 (16) Å^3^
*Z* = 2
Mo *K*α radiation
μ = 1.32 mm^−1^
*T* = 298 K
0.45 × 0.40 × 0.39 mm

#### Data collection

Siemens SMART CCD area-detector diffractometer
Absorption correction: multi-scan (*SADABS*; Sheldrick, 1996 ▶) *T*_min_ = 0.587, *T*_max_ = 0.626
4732 measured reflections
2284 independent reflections
2062 reflections with *I* > 2σ(*I*)
*R*_int_ = 0.031

#### Refinement

*R*[*F* ^2^ > 2σ(*F* ^2^)] = 0.037
*wR*(*F* ^2^) = 0.096
*S* = 1.00
2284 reflections
292 parameters
2 restraints
H-atom parameters constrained
Δρ_max_ = 0.31 e Å^−3^
Δρ_min_ = −0.36 e Å^−3^
Absolute structure: Flack (1983 ▶)
Flack parameter: 0.00 (2)

### 

Data collection: *SMART* (Siemens, 1996 ▶); cell refinement: *SAINT* (Siemens, 1996 ▶); data reduction: *SAINT*; program(s) used to solve structure: *SHELXS97* (Sheldrick, 2008 ▶); program(s) used to refine structure: *SHELXL97* (Sheldrick, 2008 ▶); molecular graphics: *SHELXTL* (Sheldrick, 2008 ▶), *Mercury* (Macrae *et al.*, 2006 ▶); software used to prepare material for publication: *SHELXTL*.

## Supplementary Material

Crystal structure: contains datablock(s) I, global. DOI: 10.1107/S1600536812023872/aa2060sup1.cif

Structure factors: contains datablock(s) I. DOI: 10.1107/S1600536812023872/aa2060Isup2.hkl

Additional supplementary materials:  crystallographic information; 3D view; checkCIF report

## Figures and Tables

**Table 1:** Hydrogen-bond geometry (Å, °)

*D*—H⋯*A*	*D*—H	H⋯*A*	*D*⋯*A*	*D*—H⋯*A*
O1—H1⋯O2	0.82	1.63	2.421 (7)	163
O3—H3*C*⋯O2^i^	0.85	1.92	2.757 (6)	170
O3—H3*D*⋯O8^i^	0.85	1.82	2.658 (8)	170
O8—H8*C*⋯O6^ii^	0.85	1.86	2.660 (7)	157
O8—H8*D*⋯O4^iii^	0.85	2.11	2.862 (7)	148

Symmetry codes: (i) 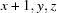; (ii) 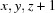; (iii) 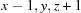.

**Fig. 1.**
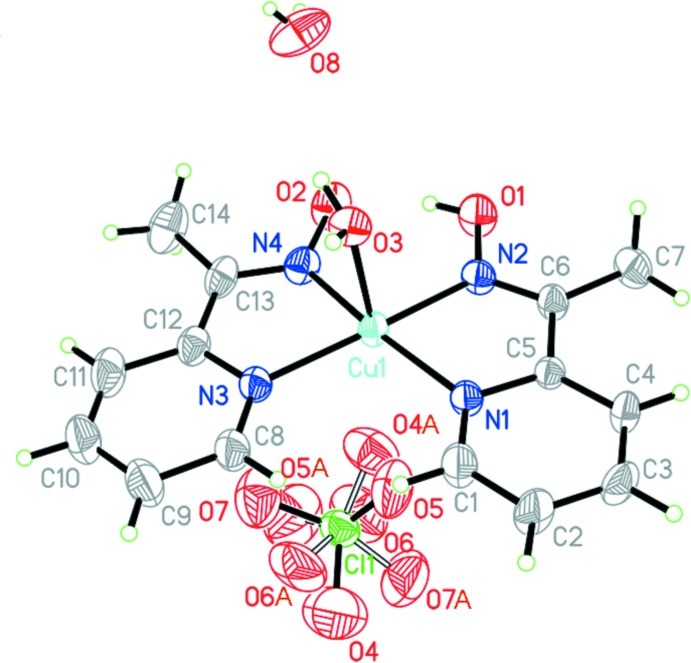
The molecular structure of the title compound with atom labels and 50% probability displacement ellipsoids for non-H atoms.

**Fig. 2.**
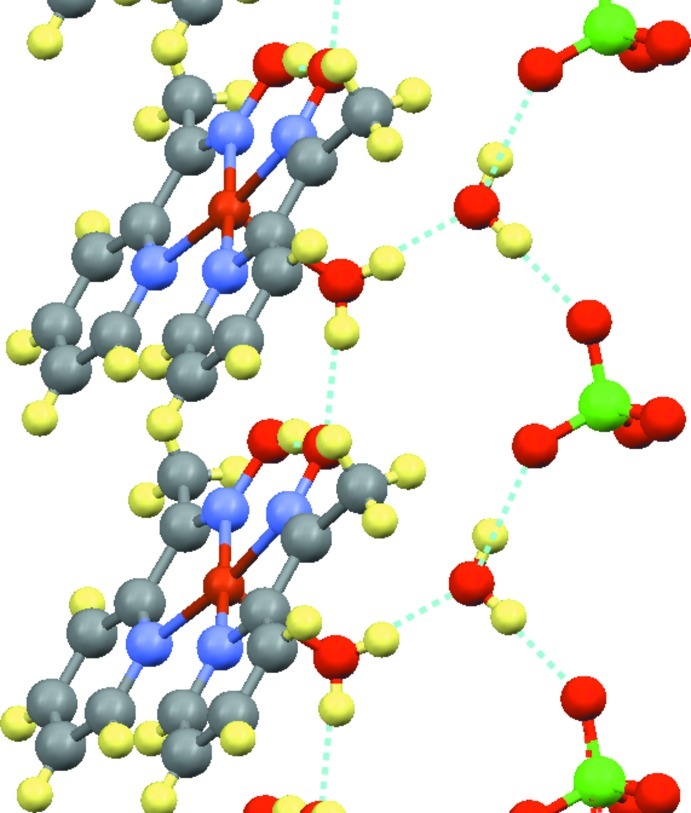
The crystal structure with hydrogen bonds shown as dashed lines.

## supplementary crystallographic information

### Comment

There is a new interest in the coordination chemistry of oximes (Davidson *et al.*, 2007; Pavlishchuk *et al.*, 2003; Chaudhuri, 2003). 2-pyridyl oximes are a subclass of oximes whose anions are versatile ligands for a variety of research objectives and have been key ligands in several areas of molecular magnetism, including single-molecule and single-chain magnets (Clerac *et al.*, 2002).

In the title complex (Fig. 1) the Cu^2+^ center is five-coordinated by N atoms from two 1-(pyridin-2-yl)ethanone oxime ligands (one of them is deprotonated) and one water molecule. The two 1-(pyridin-2-yl)ethanone oxime ligands are coordinated to copper to form two five-membered CuC_2_N_2_ rings and a strong intramolecular hydrogen bond exists between the OH group and the negatively charged oxygen of the other ligand which is shorter than reported in the literature (Qiu *et al.*, 2011; Wu *et al.* 2008). The copper atom adopts a distorted 4+1 square-pyramidal coordination mode with the distortion parameter being 0.005, which is smaller than the values reported in the literature (Qiu *et al.*, 2011; Wu *et al.*, 2008). Another water molecule and the perchlorate anion are not coordinated but they take part in the formation of H-bonds (Table 1). The perclorate O atoms are disordered between two orientations around the central Cl atom with the occupancies 0.42 (2) (O4/O7) and 0.58 (2) (O4A/O7A).

### Experimental

A solution of Cu(ClO_4_)_2_ (0.1311 g, 0.5 mmol) in H_2_O (10 ml) was added to a solution of 1-(pyridin-2-yl)ethanone oxime (0.068 g, 0.5 mmol) in MeCN (10 ml). After 0.5 h stirring, solid NaOAc (0.082 g, 1 mmol) was added slowly, and the reaction mixture was kept under magnetic stirring for another 6h. A small quantity of undissolved material was removed by filtration and the solution was left to slowly evaporate, and after one month, green crystals suitable for X-ray diffraction were obtained. (20.5%, m.p. 310-315 K). FTIR (KBr) *v* (cm^-l^): 3448 (O—H); 1597, (Cδb N); 2917, 1437, (C—H); 1157, 1177, 1260 (N—O).

### Refinement

All H atoms were placed in geometrically idealized positions [C—H 0.96 (methyl), C—H 0.93 (pyridyl) O—H 0.85 Å)and treated as riding on their parent atoms, with *U*_iso_(H) = 1.2*U*_eq_ or 1.5*U*_eq_(C), *U*_iso_(H) = 1.2*U*_eq_(O).

### Crystal data

**Table:**

[Cu(C_7_H_7_N_2_O)(C_7_H_8_N_2_O)(H_2_O)]ClO_4_·H_2_O	*F*(000) = 482
*M**_r_* = 470.32	*D*_x_ = 1.623 Mg m^−^^3^
Monoclinic, *P**c*	Mo *K*α radiation, λ = 0.71073 Å
Hall symbol: P -2yc	Cell parameters from 2353 reflections
*a* = 6.3526 (7) Å	θ = 2.5–24.1°
*b* = 15.7199 (14) Å	µ = 1.32 mm^−^^1^
*c* = 9.8235 (9) Å	*T* = 298 K
β = 101.235 (1)°	Block, green
*V* = 962.20 (16) Å^3^	0.45 × 0.40 × 0.39 mm
*Z* = 2	

### Data collection

**Table:**

Siemens SMART CCD area-detector diffractometer	2284 independent reflections
Radiation source: fine-focus sealed tube	2062 reflections with *I* > 2σ(*I*)
Graphite monochromator	*R*_int_ = 0.031
phi and ω scans	θ_max_ = 25.0°, θ_min_ = 2.5°
Absorption correction: multi-scan (*SADABS*; Sheldrick, 1996)	*h* = −7→7
*T*_min_ = 0.587, *T*_max_ = 0.626	*k* = −18→18
4732 measured reflections	*l* = −9→11

### Refinement

**Table:**

Refinement on *F*^2^	Secondary atom site location: difference Fourier map
Least-squares matrix: full	Hydrogen site location: inferred from neighbouring sites
*R*[*F*^2^ > 2σ(*F*^2^)] = 0.037	H-atom parameters constrained
*wR*(*F*^2^) = 0.096	*w* = 1/[σ^2^(*F*_o_^2^) + (0.0657*P*)^2^] where *P* = (*F*_o_^2^ + 2*F*_c_^2^)/3
*S* = 1.00	(Δ/σ)_max_ = 0.001
2284 reflections	Δρ_max_ = 0.31 e Å^−^^3^
292 parameters	Δρ_min_ = −0.36 e Å^−^^3^
2 restraints	Absolute structure: Flack (1983)
Primary atom site location: structure-invariant direct methods	Flack parameter: 0.00 (2)

### Fractional atomic coordinates and isotropic or equivalent isotropic displacement parameters (Å^2^)

**Table:**

	*x*	*y*	*z*	*U*_iso_*/*U*_eq_	Occ. (<1)
Cu1	0.90524 (7)	0.78796 (3)	0.51950 (6)	0.04434 (19)	
N1	1.0620 (8)	0.8673 (3)	0.4096 (5)	0.0487 (11)	
N2	0.7645 (8)	0.8981 (3)	0.5462 (5)	0.0529 (11)	
N3	0.9979 (8)	0.6665 (3)	0.4862 (5)	0.0501 (11)	
N4	0.7118 (8)	0.7233 (3)	0.6158 (5)	0.0559 (12)	
O1	0.6121 (8)	0.9078 (3)	0.6241 (5)	0.0749 (13)	
H1	0.5875	0.8617	0.6568	0.112*	
O2	0.5748 (8)	0.7602 (3)	0.6854 (6)	0.0786 (14)	
O3	1.1616 (7)	0.7948 (2)	0.7173 (4)	0.0574 (10)	
H3C	1.2907	0.7810	0.7164	0.069*	
H3D	1.1287	0.7759	0.7916	0.069*	
O4	0.644 (5)	0.6788 (19)	0.031 (3)	0.173 (11)	0.42 (2)
O5	0.697 (5)	0.7657 (16)	0.225 (3)	0.113 (9)	0.42 (2)
O6	0.362 (4)	0.7125 (19)	0.129 (3)	0.156 (12)	0.42 (2)
O7	0.612 (4)	0.6234 (11)	0.239 (2)	0.133 (9)	0.42 (2)
O4A	0.616 (3)	0.7497 (16)	0.277 (2)	0.120 (7)	0.58 (2)
O5A	0.421 (4)	0.6442 (16)	0.159 (2)	0.183 (10)	0.58 (2)
O6A	0.776 (3)	0.6580 (10)	0.1461 (18)	0.140 (8)	0.58 (2)
O7A	0.530 (3)	0.7535 (10)	0.0400 (16)	0.146 (7)	0.58 (2)
O8	0.0118 (15)	0.7456 (7)	0.9393 (8)	0.149 (3)	
H8C	0.1086	0.7216	0.9990	0.179*	
H8D	−0.1035	0.7468	0.9708	0.179*	
Cl1	0.5869 (3)	0.69941 (11)	0.15550 (19)	0.0719 (5)	
C1	1.2136 (12)	0.8510 (4)	0.3400 (8)	0.073 (2)	
H1A	1.2607	0.7951	0.3381	0.088*	
C2	1.3084 (13)	0.9117 (4)	0.2689 (9)	0.077 (2)	
H2	1.4162	0.8970	0.2213	0.093*	
C3	1.2380 (11)	0.9941 (4)	0.2710 (7)	0.0660 (17)	
H3	1.2958	1.0365	0.2234	0.079*	
C4	1.0826 (10)	1.0129 (3)	0.3439 (6)	0.0552 (14)	
H4	1.0358	1.0688	0.3478	0.066*	
C5	0.9947 (9)	0.9500 (3)	0.4114 (5)	0.0426 (11)	
C6	0.8256 (9)	0.9661 (3)	0.4914 (6)	0.0494 (13)	
C7	0.7378 (12)	1.0516 (4)	0.5062 (8)	0.0723 (18)	
H7A	0.7683	1.0679	0.6022	0.108*	
H7B	0.5854	1.0509	0.4730	0.108*	
H7C	0.8027	1.0916	0.4530	0.108*	
C8	1.1394 (14)	0.6393 (5)	0.4171 (8)	0.070 (2)	
H8	1.2088	0.6797	0.3724	0.084*	
C9	1.1956 (15)	0.5537 (4)	0.4048 (9)	0.085 (2)	
H9	1.2981	0.5373	0.3542	0.102*	
C10	1.0912 (16)	0.4959 (4)	0.4713 (9)	0.088 (2)	
H10	1.1217	0.4382	0.4666	0.106*	
C11	0.9453 (15)	0.5222 (4)	0.5433 (9)	0.079 (2)	
H11	0.8756	0.4826	0.5892	0.095*	
C12	0.8964 (13)	0.6082 (3)	0.5501 (6)	0.0569 (17)	
C13	0.7394 (12)	0.6412 (4)	0.6278 (8)	0.0603 (18)	
C14	0.6098 (15)	0.5892 (6)	0.7110 (10)	0.094 (3)	
H14A	0.4660	0.5828	0.6593	0.141*	
H14B	0.6064	0.6176	0.7971	0.141*	
H14C	0.6743	0.5341	0.7296	0.141*	

### Atomic displacement parameters (Å^2^)

**Table:**

	*U*^11^	*U*^22^	*U*^33^	*U*^12^	*U*^13^	*U*^23^
Cu1	0.0416 (3)	0.0393 (3)	0.0582 (3)	0.0006 (4)	0.0246 (2)	0.0052 (3)
N1	0.052 (3)	0.040 (2)	0.060 (3)	0.002 (2)	0.029 (2)	0.001 (2)
N2	0.053 (3)	0.050 (3)	0.065 (3)	0.010 (2)	0.033 (2)	0.000 (2)
N3	0.056 (3)	0.043 (2)	0.054 (3)	−0.001 (2)	0.017 (2)	0.002 (2)
N4	0.048 (3)	0.060 (3)	0.065 (3)	−0.003 (2)	0.023 (2)	0.008 (2)
O1	0.074 (3)	0.067 (3)	0.102 (3)	0.015 (2)	0.062 (3)	0.006 (2)
O2	0.059 (3)	0.080 (3)	0.111 (4)	0.003 (3)	0.051 (3)	0.021 (3)
O3	0.042 (2)	0.069 (2)	0.064 (3)	0.0020 (17)	0.0151 (19)	−0.0034 (18)
O4	0.19 (3)	0.19 (3)	0.14 (2)	0.02 (2)	0.04 (2)	−0.020 (19)
O5	0.115 (19)	0.083 (10)	0.13 (2)	−0.021 (12)	−0.001 (13)	0.023 (12)
O6	0.128 (18)	0.16 (2)	0.17 (2)	0.042 (17)	0.002 (16)	−0.028 (18)
O7	0.138 (18)	0.101 (12)	0.141 (16)	−0.011 (11)	−0.019 (13)	0.009 (10)
O4A	0.108 (14)	0.133 (15)	0.110 (13)	0.037 (11)	0.000 (8)	−0.033 (11)
O5A	0.18 (2)	0.172 (18)	0.198 (19)	−0.056 (18)	0.038 (16)	0.016 (16)
O6A	0.143 (13)	0.147 (13)	0.135 (14)	0.077 (11)	0.041 (10)	−0.022 (10)
O7A	0.187 (16)	0.123 (11)	0.120 (11)	0.013 (11)	0.006 (11)	0.038 (9)
O8	0.124 (6)	0.226 (9)	0.108 (5)	0.001 (7)	0.051 (5)	0.048 (6)
Cl1	0.0737 (12)	0.0706 (10)	0.0779 (11)	0.0041 (9)	0.0309 (9)	−0.0088 (8)
C1	0.085 (5)	0.049 (3)	0.102 (6)	0.009 (3)	0.057 (4)	0.009 (3)
C2	0.079 (5)	0.066 (4)	0.107 (5)	0.002 (4)	0.067 (4)	0.017 (4)
C3	0.069 (4)	0.062 (4)	0.075 (4)	−0.010 (3)	0.035 (3)	0.020 (3)
C4	0.059 (4)	0.039 (3)	0.069 (4)	−0.003 (2)	0.017 (3)	0.012 (2)
C5	0.045 (3)	0.037 (2)	0.047 (3)	0.000 (2)	0.015 (2)	0.002 (2)
C6	0.050 (3)	0.041 (3)	0.063 (3)	0.006 (2)	0.023 (3)	0.003 (2)
C7	0.080 (5)	0.058 (3)	0.086 (4)	0.025 (3)	0.033 (4)	−0.001 (3)
C8	0.090 (6)	0.048 (4)	0.082 (5)	0.001 (4)	0.040 (4)	0.005 (3)
C9	0.101 (6)	0.054 (4)	0.107 (6)	0.011 (4)	0.039 (5)	−0.006 (4)
C10	0.119 (7)	0.044 (3)	0.099 (5)	0.000 (4)	0.015 (5)	−0.005 (4)
C11	0.092 (6)	0.047 (3)	0.093 (6)	−0.019 (4)	0.004 (5)	0.009 (3)
C12	0.060 (3)	0.047 (3)	0.059 (4)	−0.010 (3)	0.000 (3)	0.006 (3)
C13	0.054 (4)	0.055 (4)	0.068 (4)	−0.012 (3)	0.003 (3)	0.018 (3)
C14	0.087 (5)	0.083 (5)	0.119 (7)	−0.025 (4)	0.035 (5)	0.035 (5)

### Geometric parameters (Å, º)

**Table:**

Cu1—N4	1.973 (5)	C1—C2	1.387 (8)
Cu1—N2	1.989 (4)	C1—H1A	0.9300
Cu1—N1	2.033 (5)	C2—C3	1.372 (9)
Cu1—N3	2.043 (5)	C2—H2	0.9300
Cu1—O3	2.282 (4)	C3—C4	1.360 (8)
N1—C1	1.311 (8)	C3—H3	0.9300
N1—C5	1.370 (7)	C4—C5	1.369 (7)
N2—C6	1.291 (7)	C4—H4	0.9300
N2—O1	1.355 (6)	C5—C6	1.472 (7)
N3—C8	1.300 (9)	C6—C7	1.472 (7)
N3—C12	1.345 (7)	C7—H7A	0.9600
N4—C13	1.305 (7)	C7—H7B	0.9600
N4—O2	1.340 (6)	C7—H7C	0.9600
O1—H1	0.8200	C8—C9	1.403 (10)
O3—H3C	0.8500	C8—H8	0.9300
O3—H3D	0.8500	C9—C10	1.364 (12)
O4—Cl1	1.38 (2)	C9—H9	0.9300
O5—Cl1	1.36 (3)	C10—C11	1.336 (13)
O6—Cl1	1.42 (3)	C10—H10	0.9300
O7—Cl1	1.438 (19)	C11—C12	1.392 (9)
O4A—Cl1	1.414 (18)	C11—H11	0.9300
O5A—Cl1	1.371 (18)	C12—C13	1.463 (11)
O6A—Cl1	1.385 (13)	C13—C14	1.509 (9)
O7A—Cl1	1.407 (13)	C14—H14A	0.9600
O8—H8C	0.8501	C14—H14B	0.9600
O8—H8D	0.8500	C14—H14C	0.9600
			
N4—Cu1—N2	92.72 (19)	C1—C2—H2	121.1
N4—Cu1—N1	170.52 (19)	C4—C3—C2	119.1 (5)
N2—Cu1—N1	79.4 (2)	C4—C3—H3	120.5
N4—Cu1—N3	79.7 (2)	C2—C3—H3	120.5
N2—Cu1—N3	170.2 (2)	C3—C4—C5	120.3 (5)
N1—Cu1—N3	107.58 (19)	C3—C4—H4	119.9
N4—Cu1—O3	91.35 (19)	C5—C4—H4	119.9
N2—Cu1—O3	96.39 (18)	C4—C5—N1	121.5 (5)
N1—Cu1—O3	94.67 (18)	C4—C5—C6	122.9 (5)
N3—Cu1—O3	90.01 (17)	N1—C5—C6	115.6 (4)
C1—N1—C5	117.1 (5)	N2—C6—C5	113.0 (4)
C1—N1—Cu1	130.0 (4)	N2—C6—C7	124.4 (5)
C5—N1—Cu1	112.9 (3)	C5—C6—C7	122.6 (5)
C6—N2—O1	116.6 (4)	C6—C7—H7A	109.5
C6—N2—Cu1	119.1 (4)	C6—C7—H7B	109.5
O1—N2—Cu1	124.2 (4)	H7A—C7—H7B	109.5
C8—N3—C12	117.6 (5)	C6—C7—H7C	109.5
C8—N3—Cu1	129.9 (4)	H7A—C7—H7C	109.5
C12—N3—Cu1	112.5 (4)	H7B—C7—H7C	109.5
C13—N4—O2	118.2 (5)	N3—C8—C9	125.0 (7)
C13—N4—Cu1	117.9 (5)	N3—C8—H8	117.5
O2—N4—Cu1	123.3 (4)	C9—C8—H8	117.5
N2—O1—H1	109.5	C10—C9—C8	116.3 (8)
Cu1—O3—H3C	120.6	C10—C9—H9	121.9
Cu1—O3—H3D	117.4	C8—C9—H9	121.9
H3C—O3—H3D	108.6	C11—C10—C9	119.9 (6)
H8C—O8—H8D	108.5	C11—C10—H10	120.0
O5—Cl1—O4	115.1 (18)	C9—C10—H10	120.0
O5A—Cl1—O6A	112.7 (14)	C10—C11—C12	120.7 (7)
O5A—Cl1—O7A	108.9 (13)	C10—C11—H11	119.7
O6A—Cl1—O7A	108.6 (11)	C12—C11—H11	119.6
O5A—Cl1—O4A	108.1 (14)	N3—C12—C11	120.5 (8)
O6A—Cl1—O4A	110.2 (10)	N3—C12—C13	116.1 (5)
O7A—Cl1—O4A	108.4 (11)	C11—C12—C13	123.4 (6)
O5—Cl1—O6	112.4 (16)	N4—C13—C12	113.4 (6)
O4—Cl1—O6	107.6 (18)	N4—C13—C14	120.4 (7)
O5—Cl1—O7	111.4 (13)	C12—C13—C14	126.2 (6)
O4—Cl1—O7	106.8 (16)	C13—C14—H14A	109.5
O6—Cl1—O7	102.6 (17)	C13—C14—H14B	109.5
N1—C1—C2	124.3 (6)	H14A—C14—H14B	109.5
N1—C1—H1A	117.9	C13—C14—H14C	109.5
C2—C1—H1A	117.9	H14A—C14—H14C	109.5
C3—C2—C1	117.8 (6)	H14B—C14—H14C	109.5
C3—C2—H2	121.1		
			
N2—Cu1—N1—C1	−179.9 (7)	C1—N1—C5—C4	−0.2 (8)
N3—Cu1—N1—C1	−7.0 (7)	Cu1—N1—C5—C4	179.6 (4)
O3—Cu1—N1—C1	84.5 (6)	C1—N1—C5—C6	−179.5 (6)
N2—Cu1—N1—C5	0.3 (4)	Cu1—N1—C5—C6	0.3 (6)
N3—Cu1—N1—C5	173.2 (4)	O1—N2—C6—C5	178.7 (5)
O3—Cu1—N1—C5	−95.3 (4)	Cu1—N2—C6—C5	1.3 (7)
N4—Cu1—N2—C6	−175.7 (5)	O1—N2—C6—C7	−1.2 (9)
N1—Cu1—N2—C6	−1.0 (5)	Cu1—N2—C6—C7	−178.6 (5)
O3—Cu1—N2—C6	92.6 (5)	C4—C5—C6—N2	179.7 (5)
N4—Cu1—N2—O1	7.2 (5)	N1—C5—C6—N2	−1.0 (7)
N1—Cu1—N2—O1	−178.1 (5)	C4—C5—C6—C7	−0.4 (8)
O3—Cu1—N2—O1	−84.5 (5)	N1—C5—C6—C7	178.9 (6)
N4—Cu1—N3—C8	178.0 (7)	C12—N3—C8—C9	−0.4 (12)
N1—Cu1—N3—C8	4.3 (7)	Cu1—N3—C8—C9	177.8 (6)
O3—Cu1—N3—C8	−90.6 (7)	N3—C8—C9—C10	0.0 (13)
N4—Cu1—N3—C12	−3.7 (4)	C8—C9—C10—C11	−0.2 (13)
N1—Cu1—N3—C12	−177.5 (4)	C9—C10—C11—C12	0.7 (12)
O3—Cu1—N3—C12	87.6 (4)	C8—N3—C12—C11	0.9 (10)
N2—Cu1—N4—C13	−179.8 (5)	Cu1—N3—C12—C11	−177.6 (5)
N3—Cu1—N4—C13	6.4 (5)	C8—N3—C12—C13	179.5 (7)
O3—Cu1—N4—C13	−83.3 (5)	Cu1—N3—C12—C13	1.0 (7)
N2—Cu1—N4—O2	−9.3 (5)	C10—C11—C12—N3	−1.1 (11)
N3—Cu1—N4—O2	176.9 (5)	C10—C11—C12—C13	−179.6 (7)
O3—Cu1—N4—O2	87.2 (5)	O2—N4—C13—C12	−178.6 (5)
C5—N1—C1—C2	−0.1 (11)	Cu1—N4—C13—C12	−7.5 (8)
Cu1—N1—C1—C2	−179.9 (6)	O2—N4—C13—C14	3.6 (10)
N1—C1—C2—C3	−0.4 (13)	Cu1—N4—C13—C14	174.7 (6)
C1—C2—C3—C4	1.1 (12)	N3—C12—C13—N4	4.1 (9)
C2—C3—C4—C5	−1.4 (10)	C11—C12—C13—N4	−177.4 (6)
C3—C4—C5—N1	1.0 (8)	N3—C12—C13—C14	−178.3 (7)
C3—C4—C5—C6	−179.7 (6)	C11—C12—C13—C14	0.3 (12)

### Hydrogen-bond geometry (Å, º)

**Table:**

*D*—H···*A*	*D*—H	H···*A*	*D*···*A*	*D*—H···*A*
O1—H1···O2	0.82	1.63	2.421 (7)	163
O3—H3*C*···O2^i^	0.85	1.92	2.757 (6)	170
O3—H3*D*···O8^i^	0.85	1.82	2.658 (8)	170
O8—H8*C*···O6^ii^	0.85	1.86	2.660 (7)	157
O8—H8*D*···O4^iii^	0.85	2.11	2.862 (7)	148

Symmetry codes: (i) *x*+1, *y*, *z*; (ii) *x*, *y*, *z*+1; (iii) *x*−1, *y*, *z*+1.
